# Dalbavancin as an option for treatment of *S. aureus* bacteremia (DOTS): study protocol for a phase 2b, multicenter, randomized, open-label clinical trial

**DOI:** 10.1186/s13063-022-06370-1

**Published:** 2022-05-16

**Authors:** Nicholas A. Turner, Smitha Zaharoff, Heather King, Scott Evans, Toshimitsu Hamasaki, Thomas Lodise, Varduhi Ghazaryan, Tatiana Beresnev, Todd Riccobene, Rinal Patel, Sarah B. Doernberg, Urania Rappo, Vance G. Fowler, Thomas L. Holland, on behalf of the Antibacterial Resistance Leadership Group (ARLG)

**Affiliations:** 1 Division of Infectious Diseases, Duke University School of Medicine, Durham, NC USA; 2 Duke Clinical Research Institute, Durham, NC USA; 3 Population Health Sciences and Division of General Internal Medicine, Duke University School of Medicine, Durham, NC USA; 4 Durham VA Health Care System, Health Services Research and Development, Center of Innovation to Accelerate Discovery and Practice Transformation (ADAPT), Durham, NC USA; 5 The Biostatistics Center and Department of Biostatistics and Bioinformatics, Milken Institute School of Public Health, George Washington University, Rockville, MD USA; 6 Department of Pharmacy Practice, Albany College of Pharmacy and Health Sciences, Albany, NY USA; 7 Division of Microbiology and Infectious Diseases (DMID), National Institutes of Health (NIH), Bethesda, MD USA; 8 AbbVie, Madison, NJ USA; 9 Division of Infectious Diseases, Department of Medicine, University of California, San Francisco, San Francisco, CA USA; 10 BiomX, Inc, Branford, CT USA

**Keywords:** *Staphylococcus aureus*, Bacteremia, Right-sided endocarditis, Dalbavancin, Randomized controlled trial

## Abstract

**Background:**

*Staphylococcus aureus* bacteremia is a life-threatening infection and leading cause of infective endocarditis, with mortality rates of 15–50%. Treatment typically requires prolonged administration of parenteral therapy, itself associated with high costs and potential catheter-associated complications. Dalbavancin is a lipoglycopeptide with potent activity against *Staphylococcus* and a long half-life, making it an appealing potential therapy for *S. aureus* bacteremia without the need for durable central venous access.

**Methods:**

DOTS is a phase 2b, multicenter, randomized, assessor-blinded, superiority, active-controlled, parallel-group trial. The trial will enroll 200 adults diagnosed with complicated *S. aureus* bacteremia, including definite or possible right-sided infective endocarditis, who have been treated with effective antibiotic therapy for at least 72 h (maximum 10 days) and with subsequent clearance of bacteremia prior to randomization to study treatment. Subjects will be randomized 1:1 to complete their antibiotic treatment course with either two doses of dalbavancin on days 1 and 8, or with a total of 4–8 weeks of standard intravenous antibiotic therapy. The primary objective is to compare the Desirability of Outcome Ranking (DOOR) at day 70 for patients randomized to dalbavancin versus standard of care. Key secondary endpoints include quality of life outcomes and pharmacokinetic analyses of dalbavancin.

**Discussion:**

The DOTS trial will establish whether dalbavancin is superior to standard parenteral antibiotic therapy for the completion of treatment of complicated *S. aureus* bacteremia.

**Trial registration:**

US National Institutes of Health ClinicalTrials.govNCT04775953. Registered on 1 March 2021

## Administrative information

Note: the numbers in curly brackets in this protocol refer to SPIRIT checklist item numbers. The order of the items has been modified to group similar items (see https://www.equator-network.org/reporting-guidelines/spirit-2727-statement-defining-standard-protocol-items-for-clinical-trials/).

**Table:** 

Title {1a}	Dalbavancin as an Option for Treatment of *S. aureus* Bacteremia (DOTS): A Phase 2b, Multicenter, Randomized, Open-Label, Assessor-Blinded Superiority Study to Compare the Efficacy and Safety of Dalbavancin to Standard of Care Antibiotic Therapy for the Completion of Treatment of Patients with Complicated *S. aureus* Bacteremia
Trial registration {2a and 2b}	ClinicalTrials.gov register identifier: NCT04775953
Protocol version {3}	Version 3.0, 14 October 2021
Funding {4}	National Institutes of Health (US NIH), Grant: 5UM1Al104681-09
Author details {5a}	Nicholas A. Turner^1^, Smitha Zaharoff^2^, Heather King^3,4^, Scott Evans^5^, Toshimitsu Hamasaki^5^, Thomas Lodise^6^, Varduhi Ghazaryan^7^, Tatiana Beresnev^7^, Todd Riccobene^8^, Rinal Patel^8^, Sarah B. Doernberg^9^, Urania Rappo^10^, Vance G. Fowler Jr^1,2^, Thomas L. Holland^1,2^ ^1^Division of Infectious Diseases, Duke University School of Medicine, Durham, NC ^2^Duke Clinical Research Institute, Durham, NC ^3^Population Health Sciences and Division of General Internal Medicine, Duke University School of Medicine, Durham, NC ^4^Durham VA Health Care System, Health Services Research and Development, Center of Innovation to Accelerate Discovery and Practice Transformation (ADAPT), Durham, NC ^5^The Biostatistics Center and Department of Biostatistics and Bioinformatics, Milken Institute School of Public Health, George Washington University, Rockville, MD ^6^Department of Pharmacy Practice, Albany College of Pharmacy and Health Sciences, Albany, NY ^7^Division of Microbiology and Infectious Diseases (DMID), National Institutes of Health (NIH), Bethesda, MD ^8^AbbVie, Madison, NJ ^9^Division of Infectious Diseases, Department of Medicine, University of California, San Francisco, San Francisco, CA ^10^BiomX, Inc, Branford, CT
Name and contact information for the trial sponsor {5b}	National Institute of Allergy and Infectious Diseases (NIAID) Bethesda, MD 20892 USA
Role of sponsor {5c}	The study sponsor is the NIAID. The Principal Investigator and research team (authors) are responsible for study design, data management, analysis, and writing of any eventual publications.

## Introduction

### Background and rationale {6a}

*Staphylococcus aureus* is a leading cause of bacteremia and infective endocarditis, both of which carry mortality rates as high as 15–50% [1–3]. Current standard of care for complicated bacteremia and infective endocarditis requires 4–6 weeks of intravenous (IV) antibiotic therapy, generally requiring placement of a central venous catheter [4]. Infectious complications often require operative intervention, prolonged hospitalizations, and sometimes ongoing care in a nursing home, rehabilitation facility, or long-term care facility. Central venous catheters are also associated with increased risk of secondary central line-associated bacteremia, thrombosis, or malfunction requiring replacement [5, 6]. Finally, injection drug use both increases the risk of *S. aureus* bacteremia and may serve as a barrier to receipt of traditional outpatient parenteral antibiotic therapy [7]. Safe and effective alternative treatment strategies are needed.

Dalbavancin is a lipoglycopeptide with potent activity against Gram-positive bacteria, including *S. aureus*. Dalbavancin is proven effective in the treatment of acute bacterial skin and skin structure infections, resulting in both the US and EU approvals for this indication [8–10]. Dalbavancin’s safety profile also compares favorably with vancomycin, having lower rates of renal injury [11, 12]. With a uniquely long half-life, pharmacokinetic modeling indicates that a two-dose regimen can provide effective systemic therapy for 6 weeks [13]. Two-dose dalbavancin regimens have proven successful in small-scale trials for both catheter-associated bacteremia and osteomyelitis [14, 15]. While there are no randomized controlled trials specifically assessing the efficacy of dalbavancin for the treatment of complicated *S. aureus* bacteremia, at least 5 prior phase 2/3 clinical trials included subjects with *S. aureus* bacteremia [16]. Among the 55 evaluable subjects with bacteremia, all achieved clearance of their blood cultures with 2 doses of dalbavancin [8–10, 14, 17]. Consequently, dalbavancin is an appealing alternative option for the treatment of *S. aureus* bacteremia without the need for prolonged central venous access. We have designed a study to compare whether administration of dalbavancin versus standard therapies for *S. aureus* bacteremia can lead to superior global outcomes. To best assess global outcomes, we chose a desirability of outcome ranking (DOOR) endpoint—which has specifically been developed to simultaneously consider both the effectiveness and toxicity of treatment regimens within a single outcome [18].

### Objectives {7}

#### Primary objective

To compare the desirability of outcome ranking (DOOR) endpoint at day 70 with dalbavancin versus standard of care antibiotic therapy as completion therapy for complicated *Staphylococcus aureus* bacteremia, including right-sided endocarditis in the intention-to-treat (ITT) population.

#### Secondary objectives

To compare dalbavancin in relation to standard of care antibiotic therapy for each of the following:

1. Clinical outcomes at day 70 in the modified intention to treat (mITT) population.
2. Safety in the mITT population.
3. Individual components of the DOOR in the ITT population.

#### Exploratory objectives

Additional exploratory objectives will include:

1. Comparison of clinical outcomes of dalbavancin versus standard of care in the clinically evaluable (CE) population at days 42 and 70.
2. Comparison of DOOR endpoints of dalbavancin versus standard of care at day 42 (end of therapy) in the ITT, mITT, and CE populations.
3. Compare clinical and microbiologic outcomes of dalbavancin versus standard of care in the ITT, mITT, and CE populations at days 42 and 70.
4. Compare clinical and microbiology outcomes of dalbavancin versus standard of care within key subgroups: MSSA versus MRSA, persons who inject drugs (PWID) vs those who do not, those receiving infectious diseases consultation versus those who do not, underlying site of infection (endovascular, bone/joint, pulmonary, skin and soft tissue), subjects with immune suppression (for subgroup analysis purposes, a more moderate definition of immune suppression is relative to exclusion criterion #10 below: active hematologic malignancy expected to cause ANC <500 cells/mm^3^ lasting >7 days during the study period, chronic steroid receipt equivalent to 20 mg prednisone for >2 weeks within the past month, HIV with CD4 count of <100 cells/mm^3^), and by duration of initial bacteremia.
5. Evaluate measurement of and compare patient-reported health-related quality of life (QoL) for dalbavancin versus standard of care at days 42 and 70 in the ITT, mITT, and CE populations.
6. Characterize the population pharmacokinetic (PK) profile for dalbavancin administered via 2-dose regimen in patients with *S. aureus* bacteremia.
7. Assess patient-level and clinical covariates associated with dalbavancin pharmacokinetics in patients with *S. aureus* bacteremia.
8. Examine the association between individual plasma PK concentration profiles and clinical and microbiology outcomes at days 42 and test of cure.
9. Examine the association between individualized plasma concentration profiles and occurrence of adverse drug events, including AST/ALT elevations >3X the upper limit of normal.
10. Examine the association between individualized plasma concentration profiles and late recurrence risk among the subset of patients with osteomyelitis at 6 months.

### Trial design {8}

The study is a randomized, open-label, assessor-blinded, superiority study comparing dalbavancin to standard of care antibiotic therapy for completion of therapy in adults with complicated *S. aureus* bacteremia, including native right-sided infective endocarditis, who have cleared their bacteremia.

## Methods: participants, interventions, and outcomes

### Study setting {9}

The study is a multi-center study involving 20–25 sites and 200 subjects randomized from hospitals in the USA or Canada. A list of study sites can be obtained from ClinicalTrials.gov.

### Eligibility criteria {10}

#### Inclusion criteria

1. Written informed consent obtained from the patient or legally authorized representative before initiation of any study-specific procedures
2. Age ≥18 years at time of randomization
3. A diagnosis of complicated *S. aureus* (either methicillin-sensitive, MSSA, or methicillin-resistant, MRSA) bloodstream infection
4. Treated with effective antibiotic therapy for at least 72 hours and no more than a maximum of 10 days
5. Subsequent defervescence for at least 24 h and clearance of bacteremia from the qualifying pathogen (at Screening), with negative blood culture incubated for at least 48 hours.
6. Provider is willing to treat with either dalbavancin for two doses, or standard of care intravenous monotherapy for at least 4 and *no more than* 8 weeks from randomization.
7. Patients must be willing and able, if discharged, to return to the hospital or designated clinic for scheduled treatment, laboratory tests, or other procedures as required by the protocol.
8. According to the site primary- or sub-investigator assessment, patients must be expected to survive with appropriate antibiotic therapy and appropriate supportive care throughout the study.

#### Exclusion criteria

1. Uncomplicated bacteremia, defined as *all* of the following: exclusion of endocarditis by echocardiography; catheter-associated bacteremia with removal of catheter; no implanted prostheses; negative follow-up blood cultures drawn within 48 h of initial set; defervescence within 72 h of initiation of effective therapy; no evidence of metastatic sites of infection.
2. Infectious central nervous system events, including septic emboli, ischemic or hemorrhagic stroke, epidural abscess, or meningitis (excluding prior/unrelated central nervous system events).
3. Known or suspected left-sided endocarditis or the presence of a perivalvular abscess.
4. Planned right-sided valve replacement surgery in the first 3 days following randomization.
5. Presence of prosthetic heart valve, cardiac device (e.g., implantable cardioverter defibrillator, permanent pacemaker, valve support ring, ventricular assist device) *unless* removal is planned within 4 days post-randomization.
6. Presence of intravascular graft or material (excluding cardiac stents, inferior vena cava filters in place for >6weeks, vascular stents in place for >6 weeks, non-hemodialysis grafts in place >90 days, and hemodialysis grafts not used within the past 12 months and not previously infected) *unless* removal is planned within 4 days post-randomization.
7. Infected prosthetic joint or extravascular hardware *unless* removal is planned within 4 days post-randomization or hardware was placed >60 days before bacteremia and clinically appears uninfected.
8. Polymicrobial bacteremia unless the non-*S. aureus* organism is a contaminant [19, 20]
9. Significant hepatic insufficiency (Child-Pugh class C or AST/ALT values >5x ULN at the time of randomization).
10. Immunosuppression (defined as active chemotherapy expected to cause absolute neutrophil count <100 cells/mm^3^ lasting >7 days during the study period, bone marrow transplantation in the preceding 90 days, solid organ transplantation within prior 3 months or receipt of augmented immunosuppression for rejection within 3 months, chronic granulomatous disease, HIV with a CD4 count <50 cells/mm^3^ based on last known measure).
11. History of hypersensitivity reaction to dalbavancin or other drugs of the glycopeptide class of antibiotics.
12. Treatment with either dalbavancin or oritavancin in the 60 days prior to enrollment.
13. Infection with *S. aureus* not susceptible to dalbavancin (dalbavancin mean inhibitory concentration, MIC, > 0.25 μg/mL) or vancomycin (vancomycin MIC > 2 μg/mL).
14. Planned treatment with concomitant systemic antibacterial therapy with potential efficacy against the patient’s qualifying *S. aureus* isolate, other than that allowed in the protocol.
15. Pregnant/ nursing females.
16. Females of childbearing potential must have a negative pregnancy test within 48h of randomization and use effective contraception for trial duration.
17. Other medical or psychiatric condition that may, in the judgment of the investigator, increase the risk of study participation or interfere with interpretation of study results.
18. Unwilling or unable to follow study procedures.
19. Treatment with an investigational drug within 30 days preceding the first dose of study medication.

### Who will take informed consent? {26a}

Potential participants will be identified by site clinicians in collaboration with investigators. Clinical staff at individual study sites may pre-screen using lists within the electronic health record or a clinical microbiology laboratory alert system and refer potential subjects to the research study team. Upon identification of a potentially eligible participant, a member of the research team will conduct an initial pre-screening visit at bedside, including clinical record review, discussion with the treating team, and discussion with the potential participant. Study procedures, risks, and potential benefits will be presented by principal investigator or delegate. Participants will receive a copy of the study consent and will have the opportunity to ask questions.

In the event of a language barrier or the inability to read/write, certified medical interpreters will be employed to relay study materials in the language of choice for the participant. Additionally, study forms have been pre-translated into Spanish. In the event of cognitive impairment, dementia, or delirium, consent via legally authorized representative will be permitted.

### Additional provisions for collection and use of participant data and biological specimens {26b}

The investigators do not expect to collect or use any specimens for purposes other than those included in study consent.

### Interventions description {11a}

**Fig. 1:**
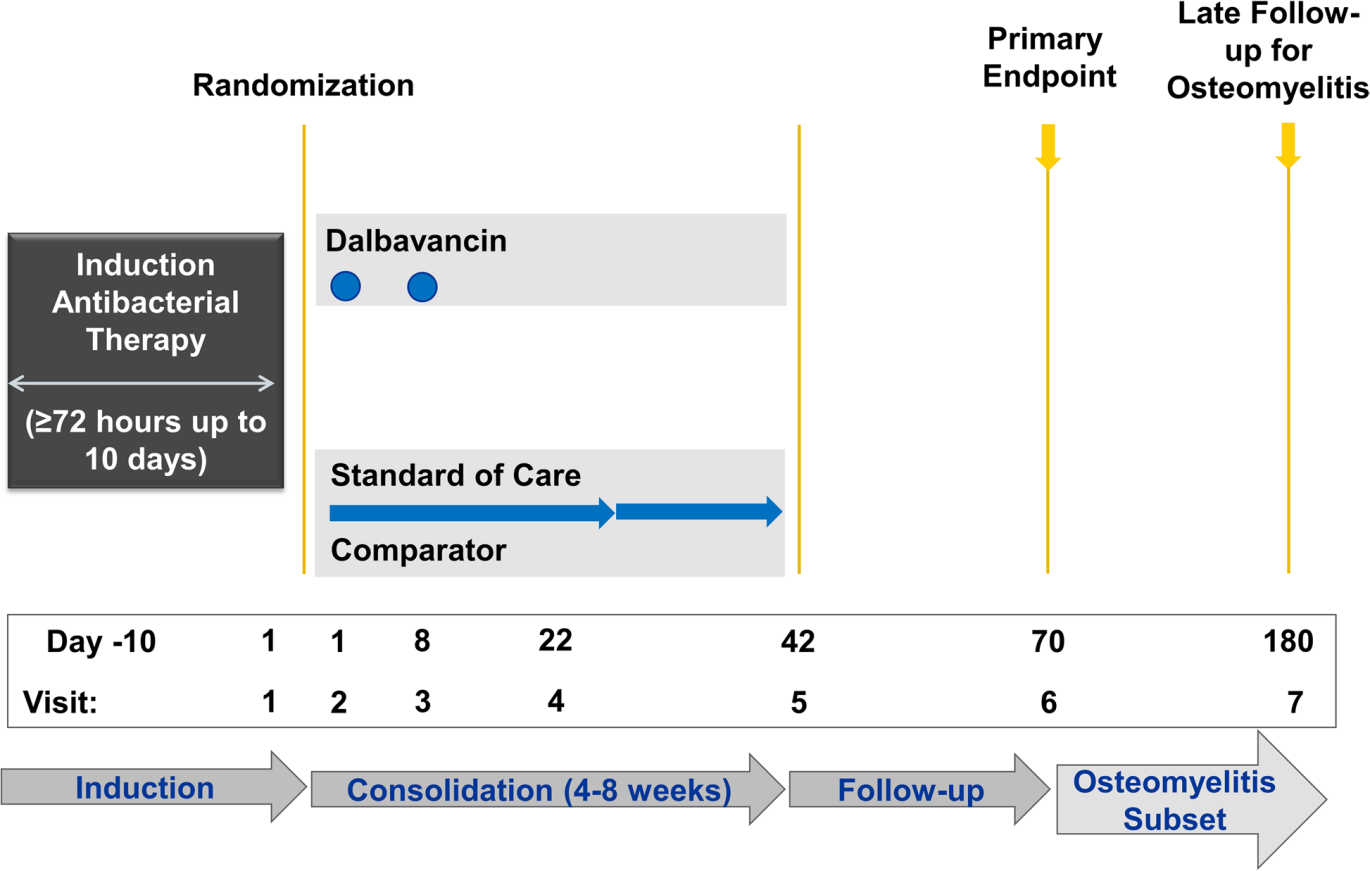
Study Schema

Initial antibacterial therapy (prior to enrollment or randomization) will be selected at the discretion of the treating clinician at participating sites. Enrolled subjects will be randomized to receive either standard of care antibiotics or dalbavancin after achieving clearance of their bacteremia. Blood cultures must be negative for at least 48 h prior to randomization, and subjects can receive no more than 10 consecutive days of effective initial therapy prior to enrollment (see Fig. 1 for study schema).

#### Explanation for the choice of comparators {6b}

Standard of care for *S. aureus* bacteremia currently includes 4–6 weeks of pathogen-directed intravenous antibiotic monotherapy. For MSSA bacteremia or right-sided endocarditis, American Heart Association (AHA) guidelines recommend nafcillin, oxacillin, or cefazolin as numerous observational studies have confirmed the superiority of anti-staphylococcal beta-lactams for this population [21–24]. At least for MSSA, vancomycin and daptomycin are considered less-preferred alternative agents, to be used only in the setting of allergy or intolerance to a first-line agent [21, 25]. For MRSA bacteremia or native-valve right-sided endocarditis, AHA guidelines recommend either vancomycin (on the basis of extensive historical experience) or daptomycin (on the basis of randomized controlled trial data) [4, 21, 26]. The dose range for daptomycin of 6–10 mg/kg IV Q24h for normal renal function is based on expert opinion, stemming in part from observational associations between higher dose ranges and improved outcomes in MRSA bacteremia [27]. To date, there is no compelling evidence of benefit from combination therapy for either *S. aureus* bacteremia or native-valve endocarditis [28, 29].

Standard of care antibiotics will be obtained from participating local hospital pharmacies in accordance with standard clinical practice and administered according to manufacturer’s instructions.

Dalbavancin will be provided by the manufacturer, Allergan (a subsidiary of Abbvie), in accordance with regulations and Good Manufacturing Practices and distributed as a study drug to participating hospitals’ investigational pharmacy staff. Un-reconstituted dalbavancin can be stored at room temperature; reconstituted drug may be stored at 2 to 8°C, or at controlled room temperature 20 to 25°C for a maximum of 48 h before use. Electronic temperature monitoring will assure against any unacceptable time/temperature excursions.

Dalbavancin will be administered in a two-dose regimen, on days 1 and 8. Dose will be determined by individual estimated creatinine clearance (by Cockcroft-Gault method): subjects with creatinine clearance ≥30 mL/min or receiving regular hemodialysis or peritoneal dialysis will receive 1500 mg over 30 (± 10) min; subjects with creatinine clearance <30 mL/min and not receiving regular hemodialysis or peritoneal dialysis will receive 1125 mg over 30 (± 10) min. A repeat serum creatinine level will be required within the 72 h prior to the day 8 dose in case of need for adjustment based on evolving renal function.

To support the proposed dosing regimen, target attainment analyses were conducted based on simulations with a recently developed population PK model for dalbavancin [13]. The most relevant predictor of clinical response for dalbavancin was determined in a neutropenic murine thigh infection model to be mean free drug daily area under the curve/minimum inhibitory concentration (fAUC/MIC) (Lepak). As a conservative assumption, the mean daily fAUC for target attainment was calculated based on dalbavancin levels on day 42. For the MIC, both the MIC_90_ of dalbavancin for *S. aureus* (0.06 mg/L) and the CLSI susceptibility breakpoint for *S. aureus* (0.25 mg/L) were used in the analyses. Results of the simulations showed target attainment on day 42 of > 99%, > 99%, and 90% for the net stasis, 1log kill, and 2log kill targets, respectively, when the *S. aureus* MIC_90_ was used. Using the CLSI breakpoint for *S. aureus*, 90% target attainment was achieved through day 42 (stasis), day 36 (1-log kill), and day 28 (2-log kill). Additionally, simulations designed to evaluate plasma concentration-time profiles suggest that a 2-dose regimen of dalbavancin of 1500 mg given on days 1 and 8 will provide plasma concentrations above the MIC_90_ of *S. aureus* for an average of 49 days after the start of therapy (internal data supplied by Allergan) [30].

#### Criteria for discontinuing or modifying allocated interventions {11b}

Duration of study drug administration will be determined at the discretion of the treating clinician and consent of the participant. Pre-specified individual halting rules include the occurrence of drug-related hypersensitivity reaction (grade 2 or higher, including anaphylaxis), new onset illness or condition which meets exclusion criteria, or the occurrence of severe adverse effects such that the investigator determines continuation of study drug not to be in the best interest of the participant.

The participant and treating physician may similarly elect to withdraw from study and/or discontinue study treatment at any time for any of the following reasons: withdrawal of consent, loss to follow-up, non-adherence, evolving clinical findings which in the judgment of the treating clinician might compromise subject safety, the subject becomes pregnant, occurrence of adverse events which in the opinion of the investigator warrant permanent discontinuation of study drug, the subject has an insufficient therapeutic response, or at the subject’s discretion.

Study withdrawal could also occur if the study or study site is terminated by sponsor for any reason.

#### Strategies to improve adherence to interventions {11c}

Initial treatment will occur in the hospital. For completion of therapy following clearance of bacteremia, subjects will be treated in accordance with local practice at participating sites. This may include outpatient parenteral antibiotic therapy for the standard of care arm or a single visit to an infusion clinic for the dalbavancin arm; alternatively some participants may require ongoing care in a hospital, nursing facility, or long-term care facility. Subject disposition and candidacy for outpatient parenteral antibiotic therapy will be left to the discretion of participating hospitals in accordance with their usual practice. As PWID will be included in the trial, we have encouraged sites to utilize substance use treatment teams where available to assist with co-morbidities that might otherwise increase risk of loss to follow-up.

#### Relevant concomitant care permitted or prohibited during trial {11d}

Other potentially effective systemic antibiotics are not permitted for the treatment of *S. aureus* after randomization. Treatment of any co-occurring infections is permitted if agents without activity against *S. aureus* bacteremia can be provided (e.g., oral vancomycin for *C. difficile*; nitrofurantoin for urinary tract infection).

Any procedures required to treat the source or metastatic complications of *S. aureus* bacteremia are not only permitted but encouraged in line with providing optimal standard of care to all participants. Eligibility is limited only by existence of the pre-specified complications listed in the “Exclusion criteria” section.

#### Provisions for post-trial care {30}

All participants will be followed to 70 days, or up to 180 days for the subset with osteomyelitis. Additionally, any subjects experiencing a post-randomization adverse event will be followed to resolution of the adverse event. Any subjects experiencing an adverse event in the course of the trial will be directed to receive appropriate care.

### Outcomes {12}

The primary outcome measure is the DOOR at day 70 [31, 32]. The clinical components of the DOOR endpoint (success/failure, adverse events, or infectious complications) will be adjudicated by an independent committee blinded to treatment assignment. Five possible DOOR endpoints are ranked as shown in Table 1. Change in health-related quality of life from baseline to day 70, as assessed by the HRQoL survey developed by the ARLG and tailored for bloodstream infections, will serve as a tie-breaker among equivalent ranks [33, 34].

**Table 1:** Primary outcome—Desirability of Outcome Ranking (DOOR) Endpoint Scoring

Rank	Alive	How many of: 1) Clinical failure 2) Infectious complication 3) SAE or AE leading to study drug discontinuation	QoL
1	Yes	0 of 3	Tiebreaker based on net change in HRQoL score from baseline
2	Yes	1 of 3	
3	Yes	2 of 3	
4	Yes	3 of 3	
5	No (Death)	Any	

Clinical success is defined as the resolution of signs and symptoms of *S. aureus* bacteremia such that no additional antibiotic therapy is required or anticipated. Infectious complications are independent of overall clinical success (e.g., one can achieve success in the end even if complications have occurred through the study course). Infectious complications include development of endocarditis, new evidence of metastatic foci of infection, relapse of bacteremia, readmission for ongoing care of the indication under study, need for additional unplanned source control procedures (e.g., abscess drainage, device removal, debridement, etc.), or change in antibiotic therapy due to inadequate clinical response.

The secondary efficacy outcome is clinical efficacy, defined by the lack of clinical failure, infectious complications, or mortality.

The secondary safety outcome is defined as the proportion of subjects who have either a serious adverse event or an adverse event leading to study drug discontinuation.

### Participant timeline {13}

Participant timeline is shown in Table 2.

**Table 2:** Schedule of events

	Induction period	Screening/enrollment	Open label treatment period				Post-treatment follow-up period		
	Visit 0 (pre-screening, day −10 to day 1)	Visit 1 (day −1 to day 1)	Visit 2 (baseline, day 1)	Visit 3 (day 8 ± 1 day)	Visit 4 (day 22 ± 2 days)	Visit 5 (day 42 ± 3 days)	Visit 6 (TOC, day 70 ± 7 days)^a^	ET^b^	Visit 7 (day 180 ± 14 days, osteomyelitis group)^a^
Informed consent		X							
Dalbavancin administration^c^			X	X					
Standard of care antibiotic therapy^c^	X	X	X (Duration 28–56 days)						
Medical history^d^		X	X			X	X	X	
Medication history^e^		X							
Randomization			X						
AEs/AESIs/SAEs			X	X	X	X	X	X	
Hematology and serum chemistry blood sampling^f^		X		X^g^	X	X			
Coagulation lab tests^f^		X							
Pregnancy test^h^		X							
PK sampling^i^			X	X	X	X	X	X	
Vital signs^j^		X	X	X^k^	X	X	X	X	X
Physical examination^l^		X	X	X	X	X	X	X	X
Echocardiogram^m^		X							
Investigator assessment of efficacy						X	X	X	X
Concomitant medications^n^		X	X	X	X	X	X	X	X
Concomitant nondrug interventions		X	X	X	X	X	X	X	X
QoL assessment^o^			X	X	X	X	X	X	X

*AEs*, adverse events; *AESIs*, adverse events of special interest; *eCRF*, electronic case report form; *ET*, early termination; *PK*, pharmacokinetic; *SAE*, serious adverse events; *TOC*, test of cure; *QoL*, quality of life

^a^Telephone visit permissible if in-person visit is not possible; in person visit still preferred

^b^Patients who prematurely discontinue therapy should have an ET Visit within 72 h

^c^All subjects will be receiving standard of care prior to randomization; after randomization, subjects will receive either dalbavancin or standard of care based on their assigned treatment group

^d^Includes targeted/pertinent medical and surgical history only

^e^A complete medication history will be completed through 30 days prior to ICF signing; an extended 60-day review will be conducted for dalbavancin and oritavancin given the long half-lives of both drugs

^f^Visit 1 hematology, coagulation lab tests (PT, PTT, and/or INR), and serum chemistry will be done in order to qualify the patient for the study, if not already collected per standard of care within 48 hours prior to randomization

^g^A serum creatinine assessment will be required within the 72 h prior to the 2nd (day 8) dalbavancin dose. Whether a serum creatinine must be repeated on day 8 will be at the discretion of the site investigator based upon stability of the serum creatinine in the preceding 72 h and whether the serum creatinine is near the threshold where dose adjustment would be necessary (e.g., near 30 mL/min)

^h^Women of childbearing potential only, if not already performed; ensure test is negative within 48 h before randomization. If the serum test results cannot be obtained before randomization, a urine pregnancy test may be used for enrollment

^i^Dalbavancin PK samples will be drawn only for subjects receiving dalbavancin. PK samples will be drawn at day 1 prior to dose, at end of infusion ± 10 min, 6 ± 2 h post end of dose, 12 ± 4 h post end of dose, 24 ± 6 h post end of dose), day 8 (prior to 2nd dose), day 22 ± 2 days (at time of clinic visit), day 42 ± 3 days, day 70 ± 7 days, and with any ET visit. Each sample must be accompanied by draw time and date

^j^Vital signs include blood pressure, respiration rate, pulse rate, and temperature

^k^Day 8 vital signs not required for subjects receiving SOC antibiotics if discharge occurs prior to day 8

^l^A physical examination (including general appearance, examination of head, eyes, ears, nose, throat, neck, skin, heart, lungs, abdomen, neurologic system, musculoskeletal system, extremities, height, and body weight) will be done at Screening (visit 1). If height or weight is not obtainable (e.g., patient is immobilized), use the last known or stated height and weight. At subsequent visits, targeted physical exams will focus on changes from prior exams and on the evaluation of newly reported symptoms

^m^Transthoracic echocardiogram or, if clinically indicated, transesophageal echocardiogram to be performed (local laboratory), unless one has been performed as standard of care for this episode of bacteremia/endocarditis

^n^All concomitant medications from screening (visit 1) through day 42 (± 3 days) (visit 5) must be recorded in the patient’s medical record and on the eCRFs. Between the day 42 visit and day 70 visit, all concomitant medications for an AE or any antibacterial therapy should be recorded in the patient’s medical record and on the eCRF

^o^QoL assessments include the ARLG Bloodstream Infection QoL Measure, the EQ-5D-5L (https://euroqol.org/eq-5d-instruments/sample-demo/), and the PROMIS Global Health Short Form (http://www.healthmeasures.net/administrator/components/com_instruments/uploads/Global%20Health%20Scale%20v1.2%2008.22.2016.pdf)

### Sample size {14}

The study is powered for a superiority comparison of the primary objective, a comparison of DOOR. The probability of a subject from the dalbavancin arm having a superior DOOR ranking relative to a subject from the standard of care arm (Wilcoxon-Mann-Whitney *U* statistic) will be used as a summary measure for the comparison. Assuming a 65% probability the dalbavancin arm will have a better DOOR than the standard of care arm, the number of participants (equally sized groups) needed to reach 90% power at 2.5% significance level for one-sided Wilcoxon-Mann-Whitney *U* test is 78 participants per arm (nQuery Advisor®, version 8.0). To account for potential loss to follow-up or dropout with approximately 12% missingness, we plan to recruit 100 per arm, i.e., 200 in total. A sample size of 200 results in at least 95.6% power.

### Recruitment {15}

We plan to conduct this study over a 2-year period. Participants will be recruited from 20–25 sites in the USA.

### Assignment of interventions: allocation

#### Sequence generation {16a}

A computer-generated, permuted block, random sequence will be used for allocation. Randomization will be stratified by the methicillin susceptibility of the pathogen (MSSA or MRSA).

#### Concealment mechanism {16b}

Participants will be randomized using Advantage eClinical, which is a single, centralized, web-based enterprise resource. Allocation concealment will be ensured, as the service will not release the randomization code until the patient has been recruited into the trial. The randomization list will be generated and kept centrally by an independent statistician who is not involved in the trial. The list will be kept confidential and allocation communicated to sites electronically via a separate online enrollment module.

#### Implementation {16c}

Eligible participants will be randomized 1:1 to either the dalbavancin or standard of care arm. The randomization process will be managed via an online enrollment module within the Advantage eClinical data management system.

## Blinding

### Who will be blinded {17a}

As an open-label trial, the participant, study site investigators, and site study teams will be aware of treatment allocation. While we considered sham infusion treatments to maintain blinding, we felt that the potential harms of unnecessary prolonged central catheter placement would have posed a potential ethical concern. Additionally, the construction of a sham infusion regimen sufficient to keep treatment ambiguous while still permitting appropriate therapeutic monitoring of vancomycin levels would have been impractical and posed risk of harm to patients.

To assure study validity, all clinical outcomes will be adjudicated by an independent committee of 4 physicians with expertise in the management of *S. aureus* bacteremia that is blinded to treatment allocation.

### Procedure for unblinding if needed {17b}

As an open label study, the site investigators, patient, and treating physician will be aware of treatment allocation.

## Data collection and management

### Plans for assessment and collection of outcomes {18a}

Demographic, clinical, laboratory, and patient-reported health-related quality of life data will be collected via electronic case report form (eCRF). Health-related quality of life forms will preferentially be interviewer-administered, with the allowance for self-administration when interviewer administration is either unfeasible or declined by the patient. Study visits will be conducted on days 1, 8, 22, 42, 70, and for the subset of subjects with osteomyelitis at day 180. The primary outcome will be determined on day 70. Secondary outcomes will be determined at day 70 for most subjects, but day 180 for outcomes specific to the subset with osteomyelitis. In-person visits are favored for day 70 and 180, but a telephone review is considered an acceptable alternative if the subject is otherwise unable to present for an in-person visit. Health-related quality of life data will be collected via the following previously developed instruments: HRQoL survey developed by the ARLG and tailored for bloodstream infections [33], EuroQoL EQ-5D-5L [35], and PROMIS Global Health Short Form v1.2 [36].

### Plans to promote participant retention and complete follow-up {18b}

Participants are free to withdraw from the study at any point, either of their own volition or at the request of their treating physician.

Study sites are encouraged to use their local existing protocols for retention of outpatient parenteral antibiotic therapy recipients, which may include call lists for appointment reminders, utilization of home visits by study teams in conjunction with home health nursing staff, and team-based management of any co-existing conditions which may affect follow-up (e.g., substance abuse treatment resources for PWID).

Logistics of administration for the second dose of dalbavancin are left to the discretion of participating sites. Options include, but are not limited to, administration via an affiliated infusion clinic, research clinic, or home health team.

Subjects who withdraw or discontinue treatment will not be replaced. Subjects withdrawing or discontinuing due to study-related adverse events will be followed to resolution of the adverse event.

Subjects will be considered clinically evaluable if they have a primary outcome assessment and do not have any missing data or major protocol violations which prevent the adjudications committee from evaluating their outcome.

Subjects withdrawing from the study will be directed back to standard care and assured that such a decision will not alter their ability to receive treatment for their bacteremia.

### Data management {19}

Electronic case report forms (eCRFs) specific to this study have been developed by Emmes Company for use by site study staff. The sponsor’s monitoring staff will either conduct site visits or remote source verification to assure veracity and completeness of data. Any discrepancies in data collection will trigger site retraining for the relevant data fields.

### Confidentiality {27}

Personal health information will be collected and stored securely within the electronic study database for up to 10 years after study completion.

### Plans for collection, laboratory evaluation, and storage of biological specimens for genetic or molecular analysis in this trial/future use {33}

Blood cultures and clinical laboratory specimens will be collected in accordance with local clinical procedures. No samples will be collected for genetic analysis.

For participants randomized to the dalbavancin arm, additional blood samples will be collected for pharmacokinetic (PK) analyses relevant to exploratory aims above. PK sampling on day 1 will be drawn prior to first dose, at the end of infusion (±10 min), 6 (±2) h after end of infusion, 12 (±4) hours post end of dose, and 24 (± 6) h post end of dose, with documented draw time and date for each sample. Subsequent PK samples will be drawn with each scheduled study visit (days 8—prior to receipt of second dose of dalbavancin, 22, 42, 70) or with any unscheduled visits triggered by an adverse event, early termination, or change in therapy. PK samples will be centrifuged and frozen on site prior to transportation to a central reference laboratory for processing (Keystone Central Laboratory).

## Statistical methods for primary and secondary outcome analysis {20a}

Analyses will be performed on the basis of the ITT principle. The primary efficacy endpoint is DOOR assessed at day 70 within the ITT population, defined as all randomized subjects regardless of whether or not they received study treatment. The primary outcome will be reported as the probability that the DOOR of a randomly selected subject from the dalbavancin arm exceeds that of a randomly selected subject from the standard of care arm (Wilcoxon-Mann-Whitney *U* statistic), along with a 95% confidence interval. In cases of equivalent DOOR scores, change from baseline quality of life will serve as tiebreaker [18]. Superiority will be considered to have been achieved if the 95% confidence interval for the probability does not cross 50%. Given the composite nature of DOOR, individual components will be analyzed and examined separately. Partial credit scoring-based analyses will also be conducted [32].

Among secondary outcomes, clinical efficacy at day 70 will be assessed for the ITT and mITT populations using a non-inferiority approach with a 20% absolute difference margin. The mITT population will consist of all patients in the ITT population who received at least one dose of study drug.

Difference in clinical failure rates between treatment groups will be calculated using generalized estimating equations with an unstructured correlation structure. Safety outcomes will be assessed by calculating risk differences for the occurrence of serious adverse events and overall adverse events at day 70 for the ITT population. Risk difference for the occurrence of each individual DOOR component will also be calculated for the ITT population at day 70.

### Interim analyses {21b}

An interim analysis for futility will be conducted after at least 50% of subjects have completed the study. Predictive interval plots will be generated for a range of assumptions, including (1) continuation of initially observed outcome trends, (2) that the alternative hypothesis is true, (3) that the null hypothesis is true, and (4) presuming best- and worst-case scenarios for remaining outcomes. By relying on prediction interval plots instead of a traditional statistical test, no power or type I error adjustment is considered for the interim analysis [37, 38].

### Methods for additional analyses (e.g., subgroup analyses) {20b}

Clinical efficacy will also be analyzed at day 42 within the ITT and mITT populations, and at day 70 for the CE population. The CE population will consist of all patients within the mITT population who have a primary outcome assessment of DOOR, without any missing data or major protocol violations which prevent the adjudications committee from evaluating outcomes. Differences in rates of clinical efficacy will be reported along with 95% confidence intervals from linear regression modeling.

DOOR will also be analyzed at day 42 among the ITT and mITT populations, as well as days 42 and 70 for the CE population. DOOR will be assessed in the same manner as with the primary outcome.

DOOR and clinical efficacy at day 42 and day 70 will be assessed in the ITT, mITT, and CE populations across the following clinically important subgroups: (1) subjects with MSSA versus MRSA, (2) PWID versus non-PWID, (3) those who received infectious diseases consultation versus those who did not, (4) by underlying site of infection (e.g., endovascular, endocarditis, bone and joint, skin and soft tissue, pulmonary), (5) by duration of bacteremia, and (6) by immune suppressed status versus non-immune suppressed (defined as active hematologic malignancy expected to cause ANC <500 cells/mm^3^ lasting >7 days during the study period, chronic steroid receipt equivalent to 20 mg prednisone for >2 weeks within the past month, of HIV with CD4 count of <100 cells/mm^3^).

Microbiologic cure will be compared between treatment groups at day 42 and day 70 within the ITT and mITT populations.

Health-related quality of life measures will be compared between treatment groups at days 42 and 70 for the ITT, mITT, and CE populations. Summary statistics will be provided for QoL scores and change from baseline QoL for each QoL instrument: ARLG Bloodstream Infection QoL measure, EQ-5D-5L, and PROMIS Global Health Short Form.

For the exploratory objectives related to pharmacokinetics, free and total dalbavancin concentration-time profiles will be graphed using box and whisker plots, with investigation of any outliers for erroneous data entry. Individual concentration-time plots will be generated on linear and log scales. Non-compartmental analysis (NCA) will be used as the initial approach for base pharmacokinetic parameter estimates. Various pharmacokinetic exposures for free and total concentration-time data will be assessed including maximum plasma concentration, time to maximum plasma concentration, as well as concentration and area under the curve (AUC) across study time points. We will also apply mixed-effects modeling to conduct a population pharmacokinetic analysis of patient-level covariates predictive of inter-individual variability in pharmacokinetic parameters. Covariates of interest will include age, gender, body size descriptors, creatinine clearance, albumin, and intravenous drug use. A final PK model will be developed using stepwise forward selection followed by stepwise backward elimination. Using the final population PK model, we will then assess the relationship between various dalbavancin exposure measures and clinical outcomes (DOOR, late recurrence, AST/ALT elevation, clinical failure) using standard exposure-response methodologies.

### Methods in analysis to handle protocol nonadherence and any statistical methods to handle missing data {20c}

The primary outcome will be analyzed on the intention to treat population. Discontinuation due to adverse event which is a potential cause for missing data is one of the components for DOOR. Key secondary outcomes, including safety and clinical efficacy endpoints, will be analyzed on the ITT and mITT populations, with mITT defined as any subjects receiving at least one dose of study drug.

To assess the potential influence of missing data on the primary outcome, the primary outcome will be analyzed using both inverse probability weighting (IPW), where a logistic regression will be used to model the probability of DOOR being complete on the basis of all available individual level covariates. Adjusted DOOR will be calculated by multiplying the inverse of the probability of completeness by the DOOR. As a sensitivity analysis, multiple imputation will also be performed by modeling missing DOOR by linear modeling. Overall effect estimates will combine the parameter estimates from 20 imputed data sets.

We will conduct analogous sensitivity analyses, using both IPW and multiple imputation, for clinical efficacy at day 70 among the ITT and mITT populations.

### Plans to give access to the full protocol, participant level-data, and statistical code {31c}

The datasets analyzed during the current study and statistical code are available from the corresponding author on reasonable request, as is the full protocol.

## Oversight and monitoring

### Composition of the coordinating center and steering committee {5d}

The Division of Microbiology and Infectious Diseases (DMID) within the NIH serves as the overall study sponsor, responsible for trial conduct and safety oversight. A DMID Clinical Research Operations and Management Support (CROMS) team will conduct site training, periodic monitoring, and close of study visits to assure proper adherence to trial protocol and research standards. Monitoring visits will include periodic review of data submission forms, source data verification, adverse event reporting, and consent documentation.

### Composition of the data monitoring committee, its role, and reporting structure {21a}

Adverse event data will be monitored by an independent data safety monitoring board (DSMB). The study may be halted by the DSMB if more than one subject death is suspected to be related to dalbavancin, or if 5 or more subjects are suspected to have developed drug-induced liver injury related to dalbavancin, or if 5 or more subjects experience grade 4 adverse effects related to dalbavancin that are coded in the same high-level group term per Medical Dictionary for Regulatory Activities (MedDRA) classification.

The DSMB will also be responsible for the interim analysis to be conducted at 50% enrollment and will report to the trial management group whether there is evidence of futility at that time. The trial will not be halted early for any evidence of superiority at the interim analysis.

### Adverse event reporting and harms {22}

Adverse events (AEs) will be monitored from time of first study dose following randomization (whether in standard of care or investigational arm) through day 70. Any AEs occurring during the study period will be followed to resolution or stabilization. AE screening and identification will be undertaken with each study visit, or may occur with any unscheduled visit initiated by the subject. Severity and causality will be assessed by the site Principal Investigator or their specified delegate.

Adverse events will be classified in accordance with the Common Terminology Criteria for Adverse Events (CTCAE), version 5.0. All events grade 3 or higher will be captured and reported. Additionally, the following adverse events of special interest (AESIs) will be captured and reported regardless of severity grade: allergic reaction, catheter related infection, vascular access complication, infusion site extravasation, infusion related reaction, alanine aminotransferase/aspartate aminotransferase increases, and acute renal injury. AESIs were selected on the basis of known or suspected associations with central venous access, intravenous antibiotic receipt, and serious or common adverse effects of included study drugs (for both standard of care and investigational arms). Study eCRFs include questions to solicit the presence of AESIs at each study visit.

All documented adverse events will be reported in any eventual trial publication and/or supplemental materials as AEs are in fact a key component of primary, secondary, and exploratory trial objectives.

### Frequency and plans for auditing trial conduct {23}

To assure correct application of inclusion/exclusion criteria across a large number of sites relative to total enrollment, each site is required to review its first 3 enrolled subjects with the overall principal investigator and/or delegate.

The CROMS team will conduct periodic site visits through trial conduct, including review of data submission forms, source data verification, adverse event reporting, and consent documentation.

### Plans for communicating important protocol amendments to relevant parties (e.g., trial participants, ethical committees) {25}

Any protocol modifications will be submitted for review by participating institutional review boards (IRBs) for approval prior to implementation. Should any amendment alter study conduct for participants, participants will be notified of the changes and will be asked to sign an updated consent form.

## Dissemination plans {31a}

Study results will be reported in accordance with Consolidated Standards of Reporting Trials (CONSORT) guidelines for randomized controlled trials. This trial was registered at clinicaltrials.gov prior to any enrollment. Results will be submitted to clinicaltrials.gov within 1 year of study completion. The authors also plan to submit study results for publication in a peer-reviewed scientific journal and/or present at relevant conferences. No PHI will be revealed in any publication or presentation.

Authorship will follow guidelines set by the International Committee of Medical Journal Editors. That is, only those who made a substantial contribution to study design, conception, data acquisition, analysis, and interpretation, participated in drafting or revising the manuscript, gave final approval for publication, and agree to accountability for the work submitted will be offered authorship. No professional writers will be used.

In addition to this protocol manuscript, the full protocol will be made available in its active form via clinicaltrials.gov.

## Discussion

At present, standard of care for complicated *S. aureus* bacteremia generally involves 4–6 weeks of intravenous antibiotic therapy. Long-term intravenous access for antibiotic therapy is associated with increased risk of complications, including catheter associated infection or thrombosis. Additionally, durable venous access may be particularly problematic for PWID—a population also at increased risk for *S. aureus* bacteremia. Consequently, there is strong clinical interest in treatment strategies that may avoid placement of intravascular access devices.

Due to its long half-life, dalbavancin offers an appealing alternative treatment option requiring just two infusions separated by 1 week in time. If at least similarly effective to current standard of care in treating complicated *S. aureus* bacteremia, dalbavancin could help to reduce the risks of long-term intravenous infusions and dramatically simplify treatment logistics—presumably offering a more favorable treatment strategy from the patient perspective as well.

The Dalbavancin as an Option for Treatment of *S. aureus* Bacteremia (DOTS) trial aims to assess whether dalbavancin is associated with more desirable clinical outcomes than current standard of care therapy. By using a DOOR approach for the primary endpoint, we hope to achieve an integrative assessment of outcomes incorporating not just clinical success, but also rates of infectious complications and adverse events which are especially relevant to patients.

## Trial status

The trial enrolled its first subject on 22 April 2021 and remains active as of the time of printing, with anticipated completion date April 2023.
